# The seventh international RASopathies symposium: Pathways to a cure—expanding knowledge, enhancing research, and therapeutic discovery

**DOI:** 10.1002/ajmg.a.62716

**Published:** 2022-03-09

**Authors:** Maria I. Kontaridis, Amy E. Roberts, Lisa Schill, Lisa Schoyer, Beth Stronach, Gregor Andelfinger, Yoko Aoki, Marni E. Axelrad, Annette Bakker, Anton M. Bennett, Alberto Broniscer, Pau Castel, Caitlin A. Chang, Lukas Cyganek, Tirtha K. Das, Jeroen den Hertog, Emilia Galperin, Shruti Garg, Bruce D. Gelb, Kristiana Gordon, Tamar Green, Karen W. Gripp, Maxim Itkin, Maija Kiuru, Bruce R. Korf, Jeff R. Livingstone, Alejandro López‐Juárez, Pilar L. Magoulas, Sahar Mansour, Theresa Milner, Elisabeth Parker, Elizabeth I. Pierpont, Kevin Plouffe, Katherine A. Rauen, Suma P. Shankar, Shane B. Smith, David A. Stevenson, Marco Tartaglia, Richard Van, Morgan E. Wagner, Stephanie M. Ware, Martin Zenker

**Affiliations:** ^1^ Department of Biomedical Research and Translational Medicine Masonic Medical Research Institute Utica New York USA; ^2^ Division of Cardiology, Department of Medicine, Beth Israel Deaconess Medical Center Harvard Medical School Boston Massachusetts USA; ^3^ Department of Biological Chemistry and Molecular Pharmacology Harvard Medical School Boston Massachusetts USA; ^4^ Department of Cardiology Boston Children's Hospital Boston Massachusetts USA; ^5^ Division of Genetics, Department of Pediatrics Boston Children's Hospital Boston Massachusetts USA; ^6^ RASopathies Network USA Altadena California USA; ^7^ Cardiovascular Genetics, Department of Pediatrics, Centre Hospitalier Universitaire Saint‐Justine Research Centre Université de Montréal Montréal Canada; ^8^ Department of Medical Genetics Tohoku University School of Medicine Sendai Japan; ^9^ Section of Psychology, Department of Pediatrics Baylor College of Medicine Houston Texas USA; ^10^ Children's Tumor Foundation New York New York USA; ^11^ Yale Center for Molecular and Systems Metabolism Yale University School of Medicine New Haven Connecticut USA; ^12^ Division of Hematology‐Oncology UPMC Children's Hospital of Pittsburgh Pittsburgh Pennsylvania USA; ^13^ Department of Biochemistry and Molecular Pharmacology NYU Grossman School of Medicine New York New York USA; ^14^ Department of Medical Genetics BC Women and Children's Hospital Vancouver British Columbia Canada; ^15^ Stem Cell Unit, Clinic for Cardiology and Pneumology University Medical Center Göttingen Göttingen Germany; ^16^ Department of Cell, Developmental, and Regenerative Biology Icahn School of Medicine at Mount Sinai New York New York USA; ^17^ Hubrecht Institute‐KNAW and University Medical Center Utrecht Utrecht The Netherlands; ^18^ Institute Biology Leiden Leiden University Leiden The Netherlands; ^19^ Department of Molecular and Cellular Biochemistry University of Kentucky Lexington Kentucky USA; ^20^ Division of Neuroscience & Experimental Psychology, Faculty of Biology, Medicine and Health, School of Biological Sciences, Royal Manchester Children's Hospital, Central Manchester University Hospitals NHS Foundation Trust, Manchester Academic Health Sciences Centre University of Manchester & Child & Adolescent Mental Health Services Manchester UK; ^21^ Mindich Child Health and Development Institute and the Departments of Pediatrics and Genetics and Genomic Sciences Icahn School of Medicine at Mount Sinai New York New York USA; ^22^ Lymphovascular Medicine, Dermatology Department St. George's University London UK; ^23^ Division of Interdisciplinary Brain Sciences, Department of Psychiatry and Behavioral Sciences Stanford University School of Medicine Stanford California USA; ^24^ Department of Genetics AI duPont Hospital for Children Wilmington Delaware USA; ^25^ Center for Lymphatic Disorders, Department of Radiology University of Pennsylvania Perelman School of Medicine Philadelphia Pennsylvania USA; ^26^ Department of Dermatology, Department of Pathology & Laboratory Medicine University of California Davis Sacramento California USA; ^27^ Department of Genetics University of Alabama at Birmingham Birmingham Alabama USA; ^28^ Igia Pharmaceuticals Florence Kentucky USA; ^29^ Department of Health and Biomedical Sciences University of Texas Rio Grande Valley Texas USA; ^30^ Department of Molecular and Human Genetics, Baylor College of Medicine Texas Children's Hospital Houston Texas USA; ^31^ Molecular and Clinical Sciences Institute St George's University London UK; ^32^ South West Thames Regional Genetics Service St George's NHS Foundation Trust London UK; ^33^ CFC International Stafford Virginia USA; ^34^ Division of Clinical Behavioral Neuroscience, Department of Pediatrics University of Minnesota Minneapolis Minnesota USA; ^35^ RASopathies Network Ottawa Ontario Canada; ^36^ Department of Pediatrics, Division of Genomic Medicine, MIND Institute University of California Davis Sacramento California USA; ^37^ Department of Ophthalmology and Vision Science, School of Medicine University of California Davis Sacramento California USA; ^38^ Neurofibromatosis Network Orlando Florida USA; ^39^ Department of Pediatrics, Division of Medical Genetics Stanford University Stanford California USA; ^40^ Genetics and Rare Diseases Research Division Ospedale Pediatrico Bambino Gesù, IRCCS Rome Italy; ^41^ Helen Diller Family Comprehensive Cancer Center University of California San Francisco California USA; ^42^ NCI RAS Initiative, Cancer Research Technology Program Frederick National Laboratory for Cancer Research Frederick Maryland USA; ^43^ Department of Pediatrics, Department of Medical and Molecular Genetics Indiana University School of Medicine Indianapolis Indiana USA; ^44^ Institute of Human Genetics, University Hospital Otto‐von‐Guericke University Magdeburg Germany

**Keywords:** cardiofaciocutaneus syndrome, Costello syndrome, neurofibromatosis, Noonan syndrome, RASopathy, signaling

## Abstract

RASopathies are a group of genetic disorders that are caused by genes that affect the canonical Ras/mitogen‐activated protein kinase (MAPK) signaling pathway. Despite tremendous progress in understanding the molecular consequences of these genetic anomalies, little movement has been made in translating these findings to the clinic. This year, the seventh International RASopathies Symposium focused on expanding the research knowledge that we have gained over the years to enhance new discoveries in the field, ones that we hope can lead to effective therapeutic treatments. Indeed, for the first time, research efforts are finally being translated to the clinic, with compassionate use of Ras/MAPK pathway inhibitors for the treatment of RASopathies. This biannual meeting, organized by the RASopathies Network, brought together basic scientists, clinicians, clinician scientists, patients, advocates, and their families, as well as representatives from pharmaceutical companies and the National Institutes of Health. A history of RASopathy gene discovery, identification of new disease genes, and the latest research, both at the bench and in the clinic, were discussed.

## 1 INTRODUCTION

The RASopathies are a group of syndromic disorders caused by germline mutations in genes found along the canonical Ras/mitogen‐activated protein kinase (Ras/MAPK) pathway, a signaling pathway critical for cellular homeostasis, cell differentiation, proliferation, and survival (Tidyman & Rauen, 2016). Although individually rare, collectively, the RASopathies comprise one of the largest families of congenital disorders worldwide. Germline pathogenic variants result in similar yet distinct syndromes, which include Noonan syndrome (NS), Noonan syndrome with multiple lentigines (NSMLs), Costello syndrome (CS), cardiofaciocutaneus syndrome (CFCS), neurofibromatosis type 1 (NF1), and other clinically related diseases, for example, Legius syndrome, Mazzanti syndrome (NS with loose anagen hair [NSLAH]), and CBL‐related and MAPK1‐related disorders. The phenotypic characteristics of the RASopathies can be severe and/or life‐threatening; they include facial abnormalities, short stature, cardiac structural and functional defects, hematopoietic defects, skeletal malformations, and certain types of cancer. These may be present at birth or develop throughout a patient's life. Historically, effective and targeted treatments for RASopathies have remained elusive, and patients are often left with limited or no options for treatment. Therefore, there is a critical need to move forward on this important area of research and the promise of finding effective treatments for RASopathies. It is clear that having a better understanding of the molecular targets for these disorders paves the way for generating more effective, more targeted, and more personalized therapies for treating patients with RASopathies. The seventh International RASopathies Symposium, held virtually on July 23–25, 2021, aimed to expand our understanding of the newest research in the field, to enhance our knowledge of potential new molecular and genetic discoveries, and to help us identify new therapeutic targets for treating these disorders. In this review, we share the proceedings of the meeting and discuss how these efforts further the groundbreaking discoveries, the clinical advancements, and the effective progress to treating these patients in the near future.

## 2 CELEBRATING THE 20TH ANNIVERSARY OF THE DISCOVERY OF *PTPN11* AS THE FIRST “RASOPATHY” GENE

Bruce Gelb, MD, and Marco Tartaglia, PhD, spoke on the history and discovery of *PTPN11*, the gene that encodes the Src homology 2 (SH2) domain‐containing protein tyrosine phosphatase 2 (SHP2), and identified as the primary cause of NS. Indeed, pathogenic variants in *PTPN11* cause greater than 50% of all cases of NS, a finding that paved the way for our understanding of RASopathies. Forty years prior, in 1962, Dr. Jacqueline Noonan was the first to describe a disorder she called “Noonan Syndrome,” a syndrome characterized by valvular pulmonary stenosis, short stature, hypertelorism, and intellectual disability. In 1994, after identifying a multigenerational family with NS, van der Burgt et al. (1994) became the first to map the location of the causal mutation of the disorder to a locus in the 5–cM region of chromosome 12, 12q24.1. In 2000, Dr. Tartaglia began a postdoctoral fellowship with Dr. Gelb, and together, in collaboration with Raju Kucherlapati, PhD, at Albert Einstein College of Medicine, Ineke van der Burgt, MD's group from the Netherlands, and Professor Michael Patton's group at St. George's, London, the group identified the NS gene. Indeed, after the successful sequencing of chromosome 12 as part of the Human Genome Project, it took these investigators only 1 h to identify *PTPN11* as the NS‐causing candidate gene (Tartaglia et al., 2001).

In partnership with Charlotte Niemeyer, PhD, Drs. Tartaglia and Gelb showed that somatic changes in *PTPN11* are causal to the majority of cases of juvenile myelomonocytic leukemia (JMML) and are also found in a minority of cases of B‐cell lineage acute lymphocytic leukemia (ALL), acute myeloid leukemia (AML), and myelodysplastic syndrome (Tartaglia, 2004; Tartaglia et al., 2003). These findings established SHP2 as the first known phosphatase to act as an oncoprotein when mutated. The specific residues involved and the types of substitutions that occurred in these NS‐associated oncogenic somatic variants of SHP2 differed from those that were NS‐associated germline variants. In fact, somatic variants associated with leukemias had higher catalytic activity of SHP2, a more pronounced hypersensitivity to cytokines and growth factors, and a higher transformation potency than those that were non‐leukemia generating NS mutations (Keilhack et al., 2005). Moreover, these mutations were found to be embryonic lethal.

Subsequent studies revealed that *PTPN11* variants are found in ~50% of all cases of NS, that individuals carrying *PTPN11* mutations are more likely to have pulmonary valve stenosis and less likely to have hypertrophic cardiomyopathy (HCM), and that all de novo *PTPN11* variants originate from the paternal copy in the presence of a paternal age effect (Tartaglia et al., 2002; Tartaglia, Cordeddu, et al., 2004). It was also discovered that the male‐to‐female ratio among these sporadic cases is 2:1. The absence of frameshift, nonsense, and splice‐site mutations in *PTPN11* also highly suggested that the pathogenic variants of NS were gain‐of‐function (GOF). Protein crystal structure analysis demonstrated that the majority of these variants were indeed located at the interactive surface between the binding domains, rendering the protein catalytically active, and with an increased binding affinity (Tartaglia, Niemeyer, et al., 2004, p. 2). This was experimentally confirmed by showing these mutations induced increased phosphatase activity in vitro (Keilhack et al., 2005; Tartaglia et al., 2006). In vivo*,* Benjamin Neel, PhD and his group created a mouse model of an NS‐causing *PTPN11* GOF mutation (Araki et al., 2004) and found that the *Ptpn11* protein product, SHP2, increased the risk of JMML (Mohi et al., 2005) and caused abnormal cardiac valvulogenesis (Araki et al., 2009).

In contrast to GOF NS variants, Drs. Gelb and Tartaglia, along with Drs. Neel and Maria Kontaridis, PhD, demonstrated that pathogenic variants causal to the allelic disorder NSML, formerly known as LEOPARD syndrome, were loss‐of‐function (LOF) and had impaired catalytic activity (Kontaridis et al., 2006; Tartaglia et al., 2006). Dr. Kontaridis's group further developed a knock‐in *Ptpn11* ^*Y279C*^ mouse model of NSML, which recapitulated nearly all of the features associated with NSML in patients, including the HCM phenotype (Marin et al., 2011). Interestingly, they found that the mechanism of these NSML‐associated *PTPN11* mutations mediated increased AKT/mTOR signaling instead of increased Ras/MAPK signaling (Marin et al., 2011), a finding previously postulated by Dr. Raynal and colleagues based on in vitro data (Edouard et al., 2010; Hanna et al., 2006). Using the mTOR inhibitor (mTORi) rapamycin, Dr. Kontaridis' group showed that they could both prevent and reverse HCM in their mouse model. This led to the idea that rapalogs may be effective in treating patients with NSML. In this regard, a patient with the NSML‐associated *PTPN11* variant Q510E, who presented with severe HCM and significantly increased AKT/mTOR activity, was treated with everolimus for 12 weeks; in that time, the infant showed significant improvement in heart function, stabilizing him enough to receive a subsequent heart transplant (Hahn et al., 2015).

Dr. Gelb also studied the neurological implications of *PTPN11* variants in a *Drosophila* model; his group demonstrated that memory impairments could be improved with alterations in conditioning protocols (Pagani et al., 2009). In addition, Alcino Silva, PhD, using Dr. Neel's *Ptpn11* ^*D16G*^ GOF NS mouse model, showed that abnormalities in neurobehavior could be improved by treatment with lovastatin (Lee et al., 2014).

Dr. Gelb also showed that one could also utilize *PTPN11‐*mutant NSML inducible pluripotent stem cell (iPSC)‐derived cardiomyocytes to recapitulate hypertrophy, the first to be used to model of a human cardiovascular disease (Carvajal‐Vergara et al., 2010). In addition, his group showed that *PTPN11‐*mutant JMML iPSCs could recapitulate myeloid abnormalities with cell autonomous effects (Mulero‐Navarro et al., 2015).

Over the last 10 years, there have been significant efforts to target SHP2 function and activity. However, direct inhibition of SHP2’s catalytic activity has not proven clinically successful. An alternate approach, however, using an allosteric peptide molecule targeting the binding of SHP2 to its signaling partners may prove to be more efficacious (Bobone et al., 2021; Fodor et al., 2018; Garcia Fortanet et al., 2016). Another promising approach aims to inhibit SHP2 binding partners and regulators, rather than SHP2 itself, as a way to modulate multiple SHP2‐dependent outputs (Yi et al., 2016).

In summary, the body of work these last 20 years on SHP2 has shown that *PTPN11* pathogenic variants cause diverse human disorders and can drive events that lead to pediatric malignancies. Furthermore, pathogenic variants can variably perturb intracellular signaling, both qualitatively and quantitatively. Importantly, the discovery of *PTPN11* in NS paved the way to the discovery of the other RASopathy genes in the pathway and to the birth of “RASopathies” as a field of research and investigation. The next steps for researchers in the coming years include better understanding of the pathophysiology associated with dysregulated SHP2 activity and function and greater insights on the dynamics associated with, and the extent to which, SHP2 signaling is dysregulated in the context of its cellular milieu.

## 3 ADVOCATE KEYNOTE: FINDING A DIAGNOSIS AND OUR VOICE AS ADVOCATES

Ms. Elisabeth Parker, a RASNet board member, is the mom of Judah and of Ezra, who has NS. She is an advocate who is passionate about creative fundraising, volunteering, speaking, and teaching yoga at RASopathies and Rare Disease events. At the meeting, she shared that when asked what 8‐year‐old Ezra wants people to know about NS, he responded, “Number one, I want people to know that when I was little, I had a tube in my belly, and number two, I had quinoa [chemo, *sic*], that I had blood draws, that I had surgeries, and that I got sick a lot.” Because Ezra did not have the typical cardiac features of NS, the path to his diagnosis was a long one. Ezra had early problems with low blood sugar, feeding, and unexplained elevated blood count. Eventually, he was diagnosed with JMML. Etched into Ms. Parker's memory is a comment from one of his care providers soon after Ezra's birth, “I've never seen a baby that looks like this.”. Experiences like this during her journey with Ezra and a firm belief that medical providers can and should communicate empathetically have made her the passionate advocate that she is today. Moreover, although physically small, Ezra's outsized personality continues to be inspirational. Armed with this, Ms. Parker actively participates in Rare Disease Day on Capitol Hill and in global Rare Disease events, has served as a board member of the RASopathies Network, organizes fundraising yoga class events, and uses social media to promote ways to support parent advocates' physical and mental health.

### 3.1 Session 1: Genes, pathways, and Genocopies

Moderated by Katherine Rauen, MD, PhD, the session started with Martin Zenker, MD, reporting on new gene candidates for the RASopathies. RASA2 and YWHAZ are negative regulators of Ras and Raf, but their precise clinical impact on the Ras/MAPK pathway needs further confirmation. CDC42 and RALA are components of a non‐canonical Ras pathway, but neither functional nor clinical data have yet sufficiently classified them as RASopathy genes (Schnabel et al., 2021). FBXW11 and TRAF7 have an uncertain link to the Ras/MAPK pathway. Hence, Noonan‐like features in some mutation carriers may be a phenocopy.

Dr. Tartaglia discussed the molecular genetics of RASopathies along with common themes and novel mechanisms in Ras signaling dysregulation. The majority of RASopathy syndrome genes have been identified by using a hypothesis‐driven approach based on gene candidacy. The collection of data on the molecular spectrum of mutations for each of these genes has contributed to the appreciation of the differential impact of RASopathy‐causing and cancer‐associated mutations on protein function and intracellular signaling. He also presented published and unpublished findings on the identification of *MAPK1* mutations as a new event underlying a novel RASopathy and on the identification of a second form of recessive NS resulting from biallelic LOF variants in *SPRED2* (Motta et al., 2020, 2021).

Yoko Aoki, MD, PhD, reviewed noncanonical GTPases RRAS2, RRAS, MRAS, and RIT1 in which pathogenic variants in these genes were identified in individuals with an NS phenotype. Overall, there are more than 30 GTPases in the Ras subfamily. RRAS, RRAS2, MRAS, and RIT1 share 48%, 55%, 57%, and 44% sequence identity with HRAS, respectively, and have an N‐ or C‐terminal extension. The sequence identity of the effector domain is very high, but hypervariable regions are unique and are predicted to have different lipid modifications.

Dr. Gelb presented a study on RASopathy genetic variation in biobanks. Rates of persons harboring a RASopathy variant in BioMe and UKBB were 1:2300 and 1:2700, respectively. Few were diagnosed with RASopathy, but a substantial fraction of individuals harboring pathogenic or likely pathogenic variants exhibited partial or full phenotypic matches to these traits (Wenger et al., 2021). Overall, there was a lower rate of RASopathy cardinal features in these individuals than expected.

### 3.2 Session 2: Novel strategies and mechanisms in RASopathies

Pau Castel, PhD, and Annette Bakker, PhD, moderated a session that included talks related to novel therapeutic strategies and mechanisms of pathogenesis in RASopathies. Lukas Cyganek, PhD, reported on the generation of cardiomyocytes as cellular models derived from iPSCs from NS patients. The iPSC technology is extremely useful in the study of RASopathies, because it allows the generation of any cellular lineage from peripheral blood mononuclear cells (PBMCs) isolated from blood or from fibroblasts isolated from skin biopsies. In his talk, Dr. Cyganek focused on iPSC‐derived cardiomyocytes from a family with NS caused by intronic mutations in the *LZTR1* gene and showed how these cells exhibit a hypertrophic phenotype that can be restored by CRISPR/Cas9‐mediated genome editing (Hanses et al., 2020).

Dr. Kontaridis described a novel pharmacological approach for treating NS‐associated HCM with the small molecule rigosertib, a Ras mimetic that inhibits both the MAPK and PI3K pathways. Using an NS knock‐in mouse model with the *Raf1* ^*L613V*^ mutation, Dr. Kontaridis showed that a 3‐week treatment schedule with rigosertib normalized chamber dimensions, posterior wall thicknesses, heart weight, and cardiomyocyte size in female mice. Results in male mice looked promising, but as yet were inconclusive. These preclinical results support the development of rigosertib for the treatment of *RAF1*‐associated hypertrophic cardiomyopathy in patients.

Emilia Galperin, PhD, presented data from her laboratory regarding the mechanism of the scaffolding protein SHOC2 in the regulation of the MAPK pathway. Dr. Galperin showed that SHOC2 orchestrates a molecular complex formed by the E3 ubiquitin ligase protein HUWE1, the AAA+ ATPase proteins PSMC5 and VCP/p97, and the de‐ubiquitinating enzyme USP7, to regulate MAPK pathway activity. USP7 plays a central role in the regulation of this complex, by promoting the inactivation of MAPK signaling. SHOC2 mutations, which have been described in individuals with NSLAH, disrupt the interaction with USP7, resulting in enhanced MAPK signaling (Wilson et al., 2021).

Caitlin Chang, MD, presented a series of clinical cases of patients that had been diagnosed with mosaic RASopathies driven by *KRAS* mutations. Somatic mosaicism occurs during embryonic development and affects specific tissue lineages depending on when and where the driver mutation occurs. Dr. Chang described eight patients with *KRAS* mutant mosaicism and their clinical features, which in several cases included embryonal tumors (rhabdomyosarcoma and Wilms tumor) and other less common symptoms, such as epilepsy, polycystic kidneys, T‐cell deficiency, and multifocal lytic bone lesions. In addition, the clinical experience from a patient treated with off‐label MEK inhibitor (MEKi) was discussed (Chang et al., 2021).

### 3.3 Session 3: Lymphatic and cardiovascular manifestations

David Stevenson, MD, and Sahar Mansour, MD, moderated a session on lymphatic problems and lymphedema in RASopathies. Kristiana Gordon, MD, discussed how pathogenic variants in RASopathy genes mediate onset of primary lymphedema, a swelling of the peripheries caused by an underlying weakness in the lymphatic system. Although the weakness is probably present at birth, the swelling may not present until childhood, adolescence, or adulthood, manifesting in lymphedema of the legs and genital area. Patients can present with leaking from the scrotum (chylous reflux) and can develop ascites, chylothoraces, or chylous pericardial effusions. Intranodal lymphangiography has also shown abnormalities of the central conducting lymphatic system, with excessive and tortuous lymphatic vessels. The Ras/MAPK pathway has an important role in lymphangiogenesis, so it is not surprising that disorders in genes that reside along this pathway affect this pathology. Indeed, it may be that up to 50% of RASopathy patients have lymphatic problems. Here, Dr. Gordon presented clinical data from the primary lymphedema clinic at St. George's Hospital, London, where she has seen over 20 cases of lymphatic disorders in patients with RASopathy. Specifically, she discussed three cases of individuals with a RASopathy who also presented with a central conducting lymphatic anomaly (CCLA). The first had a pathogenic variant in BRAF. He presented at 28 years old with lower limb lymphedema and chylothoraces, which failed to resolve despite several different therapeutic measures (e.g., thoracic duct ligation, pleurodesis, sirolimus, and octreotide). The patient responded for some time to high doses of diuretics, but sadly died following a seizure. The second patient is 24 years old with a de novo pathogenic variant in RIT1. He has bilateral lower limb lymphedema with severe genital swelling and chylous reflux. He developed marked ascites, chylothoraces, and chylous pericardial effusions, which required surgical drainage. He had multiple hospital admissions and did not respond to the usual modes of management. He was started on a MEKi, trametinib. The ascites resolved within a few weeks and the pericardial effusion improved over a few months. However, the genital edema continued. He has not had a hospital admission since starting the MEKi and is back in employment. The third case is a 10‐year‐old boy with a de novo pathogenic variant in RIT1. He has had chylothoraces in the past, treated to some degree with bilateral pleurodesis. He is currently stable, but his intranodal lymphangiography demonstrates a marked CCLA. The difficulty is knowing whether or not, and if so when, to start him on a MEKi, as there is concern his condition may continue to deteriorate.

Maxim Itkin, MD, next discussed the patterns and therapeutic options in Noonan syndrome‐related lymphatic disorders. As discussed above, patients with a RASopathy, in this case NS, often present with clinical signs of lymphatic disorders. Recent developments in lymphatic imaging techniques, such as dynamic contrast enhanced MR lymphangiography and intranodal lymphangiography, allow for new insights into the anatomy and pathophysiology of the lymphatic system. One of the main findings in almost all patients with NS is the increase in lymphatic flow rate. This can be explained by patients having either an increase in permeability of the capillaries or having a venous insufficiency and/or venous obstruction. The increase in the lymphatic flow rate results in lymphangiectasia, which in turn causes lymphatic valve insufficiency and lymphatic reflux. The clinical presentations of lymphatic reflux include chylothorax, chylous ascites, protein losing enteropathy, and genital lymphorrhea. Novel treatment approaches include interstitial/mesenteric lymphatic embolization, and percutaneous and/or surgical thoracic duct decompression, all of which provide successful treatment options for these conditions. All patients presenting with clinical symptoms of lymphatic disorders should undergo lymphatic imaging to determine possible treatment. Systematic imaging studies of the lymphatic system in NS could help to further understand the effects of the changes in the lymphatic system on the health of patients with a RASopathy (Othman et al., 2021).

Anton Bennett, PhD, presented on novel therapeutic strategies for the treatment of HCM in NSML. Up to 85% of NSML cases are caused by pathogenic variants in *PTPN11*. Utilizing the NSML *Ptpn11* ^Y279C/+^ knock‐in mouse model created by the Kontaridis laboratory, which develops HCM by 12 weeks of age, Dr. Bennett and his group found that a transmembrane SHP2 binding protein called protein‐zero related (PZR) is increased in its level of phosphorylation and binding in the hearts of these mice. When a mutation of PZR that blocks SHP2 binding is introduced into the NSML mice, HCM is blocked and AKT activity is normalized, suggesting that the interaction between PZR and SHP2 drives NSML‐associated HCM. Using dasatinib (Sprycel), an FDA approved drug for the treatment of chronic myeloid leukemia in adults and acute lymphoblastic anemia in children, Dr. Bennett showed that he could block the interaction between PZR and SHP2 and inhibit PZR tyrosyl phosphorylation in the NSML mice, even at a dose up to 100‐fold lower than that used to treat cancer patients. He further validated low toxicity and low lethality with this treatment. Importantly, echocardiograms on the mice proved that low dose dasatinib, when used before 12 weeks, could prevent the development of HCM and reduce fibrosis in the heart. He also gave the drug at 14 weeks of age, after the presentation of HCM, which reversed the hypertrophy in the cardiac muscle. The results suggest that dasatinib may be a potential therapeutic agent for HCM in NSML patients (Yi et al., 2021).

## 4 SCIENTIFIC KEYNOTE PRESENTATION

Moderated by Beth Stronach, PhD, the scientific keynote presentation by Gregor Andelfinger, MD, focused on the role of MEK and mTOR inhibition in RASopathy‐associated cardiac disease. HCM is evident in 10–20% of patients with a RASopathy and treatment options remain very limited. Infants <6 months old with RASopathy‐associated HCM can develop congestive heart failure, leading to poor survival. They face heart transplantation as the only viable option for primary treatment. The pathophysiological mechanism of RASopathy‐associated HCM is thought to be due to elevated Ras signaling, suggesting a potential benefit from Ras pathway inhibition. Indeed, elevated Ras signaling through the PI3K/AKT pathway in NSML or the Raf/MEK/ERK pathway in NS provides the rationale for considering compassionate use of mTORi or MEKi therapy, respectively (Marin et al., 2011; Wu et al., 2011). Before embarking on compassionate use treatment for direly sick patients, it is essential to thoughtfully consider the appropriate therapeutic window, drug dosage, treatment duration, outcome measures, access to, and cost of drug treatment. Comparing the strength of ERK signaling—baseline, elevated, or excessive in unaffected, RASopathy, or oncology contexts, respectively—informs the choice of MEKi dosing, with the aim to normalize signaling using a low or moderate dose of inhibitor over an extended time course, rather than to terminate signaling (and cancer) with an acute high dose therapy. Starting in 2014, compassionate use of MEKi or mTORi for progressive RAS‐HCM began to be considered. By 2018, at least three patients had been treated in pilot first‐use cases (Andelfinger et al., 2019; Hahn et al., 2015; Nelson, 2019). Increasing use over the last 2 years in more than two dozen individuals from 17 institutions on two continents has culminated in a case cohort series (for forthcoming publication), paving the way for phased trial development considerations. Dr. Andelfinger reported evidence from the treatment of this cohort, which showed improved measures of heart function and overall survival, significant avoidance of surgery, and moderate prevalence (~50%) of mostly manageable adverse effects primarily on the gastrointestinal tract and skin/hair. The positive effects of small molecule inhibitors in these RASopathy cases have excited the community, yet successful weaning from inhibitor treatment will require continued surveillance to monitor response durability and relapse. To conclude his presentation, Dr. Andelfinger recounted this cohort's experience, namely that low dose trametinib is safe and effective; it provides functional and survival benefits to patients; regression of phenotypes is seen in patients with different causal RASopathy genes/variants; and that any adverse effects must be managed with continued monitoring.

## 5 FAMILY POSTER PRESENTATIONS

Moderated by Dr. Stronach and Lisa Schill, BS, a diverse group of families shared oral stories accompanied by photos of their experiences living with a RASopathy as part of this year's virtual format. The session included individual presentations by five parents of children with RASopathy: one with CFCS, one with CS, one with NF1, two with NS, and one young adult self‐advocate with NS. Each family represented a different causal gene variant. The purpose of the session was to uncover disease impact on families, understand the patient and caregiver experience, and appreciate the humanistic side of patients and families in their natural environments. Advocates discussed both the successes and challenges in their families' journeys—through diagnosis, specialist care needs, growing awareness and benefits of advocacy, reaching milestones and often exceeding expectations.

One parent described her child's clinical and genetic diagnosis; her son had a very rare variant in *YWHAZ*, a new gene associated with CFCS. She appealed to researchers to learn more about the function of this gene, before it is too late for her son. Another parent described her child's experience taking an experimental MEKi off‐label, and how the treatment has helped improve her child's heart condition, stabilizing HCM and resolving PVS, ultimately avoiding the imminent need for heart transplantation. Each story touched on a particular need or wish based on the family's experiences, mentioning, for example, the need for vision screening; for awareness of bleeding and constipation issues; the hope for improved tumor diagnosis and earlier intervention on tumor growth; fewer side effects from current available treatments; better care coordination; and educational and emotional support. Families also emphasized the importance of community, connecting with other families to obtain perspective, learn more, support each other, and to share in their experiences. Gaining an understanding of the perspectives, unique needs, and challenges of a diverse group of patients and their families living with RASopathies will help researchers and clinicians enhance future treatments and care in the RASopathies, with the ultimate goal of improving the overall quality of life.

### 5.1 Session 4: Neurocognitive associations

Marni Axelrad, PhD, and Tamar Green, MD, moderated a session on neurocognitive associations in RASopathies. This session focused on data collected from individuals with a RASopathy and information about the brain manifestations, including data regarding brain tumors, seizures, and neurodevelopmental issues such as autism spectrum disorder (ASD). Alberto Broniscer, MD, reported preliminary results of a multi‐institutional study addressing the association of CNS cancers with non‐NF1 RASopathies. The majority of subjects in this study had NS and were younger than 18 years of age. The most commonly found cancers were low‐grade gliomas, which usually had an indolent behavior. Based on the study design, however, no conclusions could be drawn regarding the incidence of CNS cancers in this population. Additionally, no systematic screening for CNS cancers is currently recommended for children with NS.

Rene Pierpont, PhD, shared preliminary findings of her multicenter investigation of neurological and neurodevelopmental features of CFCS. Study enrollment included 138 individuals with molecularly confirmed diagnosis. An overview of the semiology and severity of seizures among study participants was provided based on information obtained from review of medical records and caregiver‐completed electronic surveys. Dr. Pierpont discussed genotype–phenotype associations with respect to seizures and neurodevelopmental outcomes. Relationships between adaptive skills of study patients and the severity of seizure burden, as well as the genotype correlation, were also examined. Her conclusion emphasized that molecular genetic testing can aid in the prediction of epilepsy in CFCS.

Domenica Battaglia, MD, focused on *BRAF* mutations in CFCS and described the electro‐clinical features, natural history of disease, long‐term outcomes, and responses to therapy. Data were longitudinal and collected from 34 patients (11 males, mean age of 15.8 ± 10.6 years, mean follow‐up of 9.2 ± 4.7 years). Of the patients, 64% (22) presented with epilepsy; 10 individuals with severe developmental and epileptic encephalopathy; and 12 had a mild epileptic phenotype. EEG characteristics of these two groups were described. A genotype–phenotype correlation linking the presence and severity of epilepsy to the nature of the structural and functional consequences of mutations was documented. Specifically, the amino acid substitutions affecting residues located within or close to the active site of the kinase were associated with a more severe epileptic phenotype, while those affecting residues placed close to the regulatory 14‐3‐3 protein binding surface were associated with mild or no epileptic phenotype. These data provide a stratification based on genotype to improve the clinical management of these patients (Battaglia et al., 2021).

Closing out this session, Shruti Garg, MBBS, MRCPsych PhD, described the social behavioral phenotype in the RASopathies, focusing on NF1, NS, and CFCS. She provided published evidence to show how the Ras/MAPK pathway may be affected in non‐syndromic autism spectrum disorder (ASD) (Geoffray, Falissard, et al., 2021). For example, 10–25% of children with NF1 present with ASD and up to 40% of them have social impairment. In research published by her group, in a cohort of children with NS (*n* = 40, 25 males), 30% met the research criteria for ASD and another 30% had partial ASD symptoms, as assessed by the ASD gold standard measures (Geoffray, Robinson, et al., 2021). The male‐to‐female ratio of ASD in NS is 1:5. Additionally, 52% of children with NS have comorbidity with ADHD. Notably, ASD symptoms are not correlated with IQ or ADHD diagnosis. Comparing ASD symptoms across the Ras/MAPK disorders, including NS, NF1, and CFCS, children with CFCS showed the most significant delay in developmental milestones, compared to the other cohorts. However, the ASD behavioral profile is similar across all RASopathy groups. Interestingly, the NF1 group showed higher levels of social communication difficulties and lower levels of restrictive repetitive behaviors, compared to NS and CFCS groups. Dr. Garg suggested there could be an issue of diagnostic overshadowing, where social impairments can often be attributed to ADHD and intellectual impairments, without a thorough assessment of ASD symptoms.

### 5.2 Session 5: Understudied clinical manifestations of RASopathies

Common manifestations of RASopathies include short stature, craniofacial features, and congenital heart defects. In this session moderated by Jeroen den Hertog, PhD, understudied manifestations of RASopathies were addressed. Karen Gripp, MD, discussed CS‐associated tumor formation. Tumors may arise in syndromic backgrounds, as well as in individuals with mosaic CS. The majority of reported CS syndromic cases have variants in HRAS residues 12 and 13, and a minority of cases have variants in other residues. All syndromic cases reported to date that develop embryonal rhabdomyosarcomas have specific mutations in residue 12, including G12S, G12C, and G12A. Moreover, all of these variants are paternally derived and display uniparental disomy (UPD) for chromosome 11, which carries the HRAS gene. HRAS mutations are commonly found in isolated cancers, with hotspots identified at residues 12, 13, and 61 in the COSMIC database (Tate et al., 2019). Yet, it is notable that the G61 variant is not typically seen in CS. Several mosaic CS cases were discussed; some showed typical CS features but harbored typical CS variants only in a small subset of cells and tissues. For example, the mother of a child with CS was found to have mosaicism for a typical CS variant, providing evidence for maternally derived CS. Several other mosaic HRAS variants with G12S mutations identified in various tumor types were also described, including cases of urothelial cell cancer and bladder cancer. Moreover, a mosaic HRAS variant with G13R mutation—which has not been reported previously in CS, but has been found in tumors—was associated with two independent embryonal rhabdomyosarcomas. In conclusion, if the HRAS mutation is present from fertilization onwards, tumor incidence depends on the specific mutation. However, if the HRAS mutation occurs post‐zygotically, tumor incidence depends on the specific mutation and on the affected tissue.

Katherine A. Rauen, MD, PhD, addressed skeletal muscle hypotonia in patients with CFCS, which manifests in the newborn period with delayed motor skills, muscle weakness, decreased muscle bulk, and suck‐swallow issues. Muscle weakness appears to improve somewhat when children grow older, but muscle bulk typically stays underdeveloped, and some children are not ambulatory. To investigate these manifestations, a mouse model for CFCS carrying a *Braf* ^*L597V*^ mutation was used (Andreadi et al., 2012). These mice have decreased skeletal muscle mass and decreased muscle strength (Maeda et al., 2021). While no apparent differences in morphology were observed, the muscle fibers were found to be significantly shorter in *Braf* ^*L597V*^ mice than in wild type mice. Whole mount immunohistochemistry of mouse embryos at E11.5 showed reduced staining using a myogenin‐specific probe, indicating inhibition of myogenesis in the mutant mice. Analysis of signaling demonstrated enhanced MEK and ERK phosphorylation and downregulation of p38 phosphorylation. Primary myoblasts were derived from wild type and *Braf* ^*L597V*^ mouse embryos; in vitro differentiation of these myoblasts was impaired in mutant cells, as compared to wild type. Treatment with a MEKi rescued differentiation of mutant myoblasts. Just as important, under these conditions, the MEKi did not significantly impact differentiation of wild type myoblasts. Taken together, the *Braf* ^*L597V*^ mouse model for CFCS develops skeletal myopathy and may be instrumental in determining the feasibility of developmentally correcting this myopathy by prenatal administration of MEKi. Moreover, it is possible that postnatal administration of MEKi can rescue aspects of the abnormal muscle phenotype in CFCS.

Maija Kiuru, MD, PhD, discussed skin and hair manifestations that are important in making clinical diagnoses of RASopathies. There is significant overlap in these features between patients with CFCS and CS, including curly hair and thickening of the palms and soles. However, there are also clear differences. For instance, absent or sparse eyebrows, darker and thicker scalp hair, and keratin‐clogged hair follicles and sweat ducts are typically only observed in CFCS (Urban et al., 2020). Conversely, papillomas on the nose and face and deep acral creases are more typical in CS. There is also an overlap in genes associated with melanoma and the RASopathies, in that mutations in *BRAF*, *NRAS,* and *NF1* in melanoma are also well‐known RASopathy‐associated genes. In patients with CFCS, a significant increase in the number of melanocytic nevi is observed (Kiuru et al., 2020). Importantly, while the number of nevi is usually the strongest risk marker of melanoma, this association for CFCS remains as yet unknown. However, protection from UV light—another risk factor in melanoma—and regular skin exams are recommended for these patients.

Suma Shankar, MD, PhD, presented an overview of ocular manifestations in RASopathies based on retrospective medical record analysis and cross‐sectional studies, particularly as it pertains to patients with CFCS, CS, and NS (Gripp et al., 2019; Pierpont et al., 2014). Eye manifestations are present in more than 90% of individuals with a RASopathy, with the most common symptoms including blurred vision, problems with stereopsis, and photophobia. Upon exam and chart review, a majority of these individuals also had strabismus, refractive errors, nystagmus, ptosis, and optic nerve anomalies, findings that are much more common in individuals with a RASopathy than in the general population (*p < 2.2E‐16*). Rarely, additional features, including keratoconus, prominent corneal nerves, retinal dystrophies, delayed visual maturation, and cortical visual impairment, were noted. Ocular features such as refractive errors, strabismus, nystagmus, and ptosis may present in infancy or later, and are amenable to correction with glasses and/or surgery (Shankar et al., 2021). Consequently, pediatric ophthalmology evaluation at the time of diagnosis, with routine biannual or annual follow‐up, is recommended to prevent amblyopia, facilitate optimal vision development, improve vision, and improve the quality of life for these individuals.

### 5.3 Session 6: Selected junior investigator presentations

Tirtha Das, PhD, presented multiple *Drosophila* models that identify RASopathy subtype‐specific differences. The *Drosophila* model has been a powerful genetic system with which to decipher fundamental cell and developmental biology questions, and it includes discovery of components of the Ras/MAPK pathway. To gain a more comprehensive understanding of how variants in genes encoding Ras/MAPK components are associated with RASopathies, he developed 13 *Drosophila* transgenic lines. Each fly line expressed a different human disease isoform associated with a RASopathy. Using these models, he developed a platform that explored tissue phenotypes, signaling pathway activity, changes in cell biology, deregulation of transcriptional targets of signaling pathways, and response to potential therapeutics (Das et al., 2021). In addition, the group performed targeted genetic modifier tests to gain insights into mechanisms promoting disease progression, as well as to validate or identify novel targets for therapeutics. Specifically, they discussed how expressing the *RAF1* ^*L613V*^ variant in developing wing tissue promoted excess wing vein formation, suggesting a GOF effect of this variant. In contrast, similar expression levels of the *RAF1* ^*D486G*^ variant inhibited wing vein formation, suggesting a potential dominant negative effect of this mutation. A multi‐panel western blot analysis of these two variants showed that both could activate markers of multiple signaling pathways. Using the newly developed CRIMIC technology, they found that the *RAF1* ^*D486G*^ variant rescued a small percentage of flies (2–5%), allowing them to reach adult stages, even in the absence of endogenous Raf function. Taken together, the group postulated that *RAF1* ^*D486G*^, previously described as a LOF variant, had a more complex function than previously known. Indeed, this *RAF1* ^*D486G*^ variant could directly or indirectly activate Ras/MAPK (and other signaling pathways), and thereby rescue loss of Ras/MAPK function. Conversely, in other contexts, the same variant can also have dominant negative effects on the same pathway (e.g., wing). In the future, using additional *Drosophila* lines (33 in total), the group plans to provide a broad overview of how RASopathy variants alter the signaling network in different tissues. These data may provide valuable insights into how pathogenic variants promote specific traits in humans, as well as enable tailored therapeutic approaches to treat them.

Alejandro López‐Juárez, PhD, discussed how abnormal myelin differentially impairs learning in models of NF1 and CS. He showed that adult *Nf1* ^*+/−*^ mice have increased oligodendrocyte (brain myelin producing cells) precursor cells with less proliferative capacity. *Nf1* ^*+/−*^ mice also showed late subnormal learning when subjected to a myelin‐regulated fine motor skill test (running on a Complex Wheel; CW). Such defects were attributed to a progressive loss of activity, without affecting capability to run. Similar results were observed in mice with myelin‐specific *Nf1* mutation, supporting the idea of a myelin‐regulated phenotype. In contrast, mice with a myelin‐specific CS‐causing mutation showed early abnormalities in the CW learning curve, but these were caused by decreases in the maximum speed achieved. In connection with previous findings on RASopathy‐associated myelin structural defects, it was suggested that parameters controlling learning in NF1 and CS are differentially regulated by myelin integrity and/or plasticity.

Richard Van, BS, and Morgan Wagner, BS, presented a biochemical analysis of RIT1, which revealed preferential interaction at the plasma membrane with RAF1 and provided evidence for therapeutic intervention of cardiac hypertrophy. Indeed, RIT1 uses a combination of specific hydrophobic residues and electrostatic interactions in its polybasic regions to associate with the plasma membrane. Interestingly, RIT1 shows preferential binding to RAF1 compared to ARAF and BRAF, which may help to explain the high incidence (~54%) of HCM in RIT1‐associated NS. In a preclinical trial using a *Rit1* mouse model of NS (Castel et al., 2019), treatment with trametinib prevented progression of cardiac hypertrophy, as determined by heart weight and myocyte size. This study provides more evidence for the use of trametinib in children with NS and RIT1‐associated HCM.

### 5.4 Session 7: Learning from each other: Perspectives on diagnostics and therapeutic interventions

Stephanie Ware, MD, PJ Brooks, PhD, Bruce Korf, MD, PhD, and Jeff R. Livingstone, PhD, held a roundtable discussion moderated by Stas Shvartsman, PhD, to discuss therapeutic interventions in the RASopathies. Dr. Ware highlighted the need for early diagnosis of patients with a RASopathy. Currently there is no “standard of care” to determine if a genetic evaluation should be triggered for babies entering the newborn intensive care unit. She also discussed the need for a more tailored approach for use of repurposed treatments in RASopathies. Many questions remain regarding treatment considerations, including type and extent of treatment for the different RASopathy syndromes and if there is a critical window in which to do so. A national registry for patients on treatment regimens that outlines their experiences would be extremely useful.

Dr. Korf discussed the need to explore both shorter term therapeutic options, such as small molecule treatments, as well as longer term options, such as gene replacement or genome editing in the RASopathies. There was also agreement on the need for a concerted effort to bring the natural history data of the RASopathies together in a centralized database.

Dr. Livingstone, CEO of Igia Pharmaceuticals, discussed the difficulty in approvals and the increased costs associated with repurposing small molecule treatments for rare diseases, in large part due to the small population size and the regulatory hurdles needed to do so. However, the RASopathies have an advantage—multiple syndromes can be treated by targeting a single pathway. He also discussed the importance of leveraging patient advocacy groups and the need for more interaction with these groups.

Dr. Brooks, from the National Center for Translational Science (NCATS), discussed the role of the NIH and NCATS in supporting basic science. He provided information on NCATS funding (https://ncats.nih.gov/funding/open) and mentioned that basket clinical trials to determine the effects of treatments on multiple diseases at the same time may be applicable to the RASopathies.

In summary, the panel viewed the importance of community efforts, including access to diagnostic testing, patient registries, definition of endpoints in clinical trials, and collaborative clinical trials, as critical to achieving success in developing and testing treatments for RASopathies. Patient advocacy groups were also viewed as crucial partners in all these efforts, facilitating and guiding the science to finding successful therapies for patients.

The symposium closed with a family panel discussion on the compassionate use of MEKi for treatment of RASopathies, which was moderated by Pilar Magoulas, MS, CGC, and Ms. Schill. This panel included parents of individuals with different RASopathies who have tried MEKi for different indications. Each of the panelists were asked questions regarding the decision to undergo treatment with MEKi, the perceived benefits and limitations, as well as the impact of the MEKi on their child's symptoms.

The first panelist was a father of an 8‐year‐old boy with NF1 who had a large plexiform neurofibroma. The family had previously tried to get enrolled in a phase 3 clinical trial for a MEKi, but was unsuccessful. Their oncologist, however, was able to prescribe trametinib off‐label through an expanded use for the treatment of plexiform neurofibromas. The child was on trametinib for approximately 2 years before switching to selumetinib, when it received FDA approval. The father expressed satisfaction with the use of the MEKi and commented that his son expressed less pain, tickling and tingling sensations, felt a difference in the texture and nature of the neurofibromas and plexiform neurofibromas, and that the plexiform neurofibroma itself had stabilized in size over time.

The second group of panelists were parents of a 9‐year‐old girl with CS who had severe HCM. She had two previous open heart surgeries to remove accessory tissue on her mitral valve when, at 8½ years of age, the parents were told that the child would need another surgery within the next 6–12 months for correction of her septal wall and aortic stenosis. It was at that time that the parents decided to try a MEKi for their child rather than go through a third open heart surgery. Their daughter has now been on a MEKi for the last 9 months and doing very well. She has not had progression of her heart disease and her echocardiograms have reportedly stabilized, whereas in the past they had progressively worsened over time. Anecdotally, they also noticed that she has had increased hair growth and height, in addition to the cardiac functional improvements.

The third panelist was a father of a 14‐year‐old boy with *PTPN11*‐associated NS and who also had worsening lymphedema in the groin area for the past 2 years. The family was told that there were no treatments available, so the father did his own research and contacted a specialist who had been studying MEKi for treatment of HCM. After clinical review of his case, reviewing the safety profile of MEKi, and weighing the benefit–risk ratios, the family and specialist initiated a MEKi treatment regimen for the child. He has been on the MEKi now for 10 months. In that time, the family has seen significant improvements in his gastrointestinal symptoms, with decreased vomiting which had occurred daily. The father also noted that they have seen improvement in pain, that the child generally “feels better,” and that he has shown an increase in height as well. They are now awaiting additional results to determine if there has been objective improvement in the lymphatic abnormalities as well.

The final panelist was a mother of a 25‐year‐old young woman with CFCS who is not currently on a MEKi, but who has expressed an interest in utilizing this treatment for her daughter and other individuals with CFCS, particularly for seizures which can cause significant morbidity. In hopes that this will be an active area of future research, the mother indicated that she would be interested in clinical trials that specifically explore the use of MEKi in individuals with CFCS, with a focus on seizure management and improvement.

Overall, all of the panelists expressed positive feedback regarding the benefits of using MEKi in their children, some of which included subjective improvements of clinical features that may not have been the primary indication for initiation of MEKi use (i.e., improved pain symptoms in those where the primary indication for using the treatment was for lymphedema or plexiform neurofibromas). While several of the panelists commented on the potential side effects of MEKi, such as sensitivity to sun and heat, skin sensitivity, and mouth sores, in their opinion, they felt that the benefits for their children, given their medical complications, far outweighed the risks. As more individuals with RASopathy receive MEKi on a compassionate use basis, the anecdotal experience of the parents and patients themselves will be crucial to understanding and ascertaining their use for future clinical trials. Indeed, we may find even more unexpected benefits to MEKi usage for the RASopathies, ones that may be even more critical and important to the quality of life of these individuals.

In summary, results from current research efforts are finally making their way to the clinic for treatment of patients with a RASopathy. The progress that is being made will have a significant impact on the lives of both the affected individual and their families. The seventh International RASopathies Symposium highlighted the research knowledge we have gained over the years that has now allowed us to find potential life‐changing and effective therapies. The hard‐fought efforts of patients, advocates, research scientists, clinicians, drug companies, and regulatory agencies have finally moved the needle forward; indeed, the time for treating RASopathies in the clinic has arrived.

## ADDITIONAL FUNDING ACKNOWLEDGMENTS

Work described herein is supported by NIH‐R01‐HL102368 and the Masonic Medical Research Institute to MIK; NIH‐R35 HL125742 to BDG.

## AUTHORS' CONTRIBUTIONS

AMB is an equity stakeholder of Igia Pharmaceuticals. PC is a founder and advisory board member of Venthera, Inc. BDG receives royalties from GeneDx; Correlegan; LabCorp; Prevention Genetics; SRA: Onconova Therapeutics; and consultancy for Day One Biopharmaceuticals. MI provides research support for Guest Group. BRK is on the medical advisory committees of GenomeMedical, SpringWorks Therapeutics, and Infixion Bioscience. MIK receives grant funding from Onconova Therapeutics.

## Data Availability

Data sharing not applicable to this article as no datasets were generated or analysed during the current study.
